# Determinants of the hCG Concentration in the Early Luteal Phase After Final Maturation of Follicles With Bolus Trigger of Recombinant hCG

**DOI:** 10.3389/fendo.2020.00137

**Published:** 2020-03-19

**Authors:** Lan N. Vuong, Toan D. Pham, Vu N. A. Ho, Tuong M. Ho, Peter Humaidan, Claus Yding Andersen

**Affiliations:** ^1^Department of Obstetrics and Gynecology, University of Medicine and Pharmacy at Ho Chi Minh City, Ho Chi Minh City, Vietnam; ^2^IVFMD, My Duc Hospital, Ho Chi Minh City, Vietnam; ^3^HOPE Research Center, My Duc Hospital, Ho Chi Minh City, Vietnam; ^4^The Fertility Clinic, Skive Regional Hospital, Skive, Denmark; ^5^Faculty of Health, Aarhus University, Aarhus, Denmark; ^6^Faculty of Health, University of Southern Denmark, Odense, Denmark; ^7^Laboratory of Reproductive Biology, Faculty of Health Science, Copenhagen University Hospital, Copenhagen University, Copenhagen, Denmark

**Keywords:** human chorionic gonadotropin, luteal phase, hormones, progesterone, *in vitro* fertilization, pharmacokinetics

## Abstract

**Introduction:** It has recently been shown that late follicular phase progesterone levels correlate well with those in the early luteal phase, and that progesterone levels before and 12 h after human chorionic gonadotropin (hCG) administration predict levels during the early luteal phase. This study investigated determinants of serum hCG levels after a bolus dose of hCG for triggering ovulation in women undergoing *in vitro* fertilization (IVF).

**Materials and Methods:** This retrospective analysis was performed on data from a prospective study of women aged 18–42 years with normal ovarian reserve receiving gonadotropin-releasing hormone (GnRH) antagonist co-treatment during ovarian stimulation with follicle-stimulating hormone (FSH) who were followed until 6 days after oocyte pick-up (OPU) in a single IVF cycle. The main outcome measures were early luteal phase serum hCG levels, and predictors of those levels.

**Results:** There was wide inter-individual variability in early phase hCG concentrations over the period from 12 h after hCG injection up to 6 days after OPU. Patients with serum hCG values in the bottom 10% had a significantly higher body mass index (BMI; *p* = 0.038) and a significantly longer duration of stimulation (*p* = 0.014) than those with higher serum hCG values. Serum progesterone levels up to the first 36 h after hCG injection were significantly higher in the low vs. higher serum hCG group, but were similar at all other time points. There was a significant correlation between serum hCG level after hCG administration and BMI (lower BMI = higher serum hCG). In a cluster analysis, patients with the lowest serum hCG and progesterone levels at 12 h after hCG injection had significantly higher BMI, and significantly lower anti-Müllerian hormone level, duration of stimulation, and number of follicles of ≥11 and ≥14 mm compared with the other three clusters.

**Conclusion:** Predictors of low serum hCG after a trigger bolus were difficult to determine, but BMI seems to be important. More detailed information on the luteal phase hormonal profile and data on predictors of hormone levels during this critical period can facilitate the development of strategies to allow individualization of the luteal phase support regimen, potentially improving IVF outcomes.

## Introduction

Human chorionic gonadotropin (hCG) is the gold standard treatment for inducing final maturation of follicles during a variety of infertility treatment modalities (1). This hormone acts as a surrogate for the mid-cycle luteinizing hormone (LH) surge in natural cycles, triggering final maturation of oocytes and ovulation, and stimulating the corpus luteum to secrete progesterone to improve endometrial receptivity (2).

Despite its widespread use, very few studies have investigated the pharmacokinetics of hCG used as a bolus trigger. The study that provided pharmacokinetic data for the registration of recombinant hCG (r-hCG) showed a relatively uniform profile for up to 12 days after administration of a single 5,000 IU subcutaneous dose in healthy male and female volunteers (3). In contrast, data from a study including two women with hypogonadotropic hypogonadism showed marked variation in the pharmacokinetic profile of subcutaneous hCG 5,000 IU between the two individuals (4). The authors suggested that this may have been due to differences in the body mass index between the patients (18.9 and 24.0 kg/m^2^); there was also a 10-year age difference between the subjects (22 vs. 32 years) (4).

Overall, currently available data indicate that hCG levels reach a critical low point around the mid-luteal phase (5, 6) and the effect of this on pregnancy outcome in patients undergoing IVF are not yet known. Luteinizing hormone activity, such as that provided by hCG, is important to ensure the continuous release of progesterone from corpora lutea, which, in turn, is essential for stimulating secretory transformation of the endometrium, facilitating successful implantation (5).

It has been shown that some women only show a modest increase in progesterone levels during the luteal phase despite a bolus trigger of hCG (7). However, whether this is due to a low concentration of hCG and lack of corpus luteum stimulation is currently unknown. Variations in hormone levels may reflect inter-individual differences in the pharmacokinetics of hCG.

We recently published the first detailed prospective study of early luteal phase hormonal profiles in women undergoing ovarian stimulation for IVF/ICSI followed by hCG trigger and a freeze-all strategy (6). The current analysis used data from that study and was performed to characterize the pharmacokinetic profile of hCG administered as a bolus for triggering ovulation in normal women and to evaluate potential characteristics associated with the time course of hCG levels during the early luteal phase in these patients.

## Materials and Methods

### Study Design

This retrospective analysis used data from a single center, prospective study conducted at IVFMD, My Duc Hospital, Ho Chi Minh City, Vietnam from June 2016 to July 2017 (NCT02798146 for original study protocol and NCT03174691 for the amended protocol with an increased sample size) (6). The study protocol and its amendment were approved by the Ethical Board of My Duc Hospital (approval numbers 01/16/ÐÐ-BVMÐand 10/17/ÐÐ-BVMÐ, dated 31 May 2016 and 10 May 2017, respectively). All patients provided written informed consent prior to enrolment in the study, which was conducted in accordance with the ICH Harmonized Tripartite Guideline for Good Clinical Practice and the ethical principles of the Declaration of Helsinki.

### Study Subjects

Full details of the study population have been described previously (6). In brief, study subjects were aged 18–42 years, had a body mass index (BMI) <28 kg/m^2^, and were undergoing IVF followed by a freeze-all cycle after hCG trigger. Other inclusion criteria included the following: normal ovarian reserve, defined as anti-Müllerian hormone [AMH] level >1.25 ng/mL or antral follicle count [AFC] ≥6, measured within the 2 months prior to starting stimulation; and GnRH antagonist co-treatment during ovarian stimulation. Patients were excluded if they had previously had a poor response (≤ 3 oocytes) or hyper-response (>20 follicles at ≥14 mm) to stimulation, if they were participating in another clinical trial, or had a chronic medical condition.

### Ovarian Stimulation, Monitoring, Trigger, and Oocyte Removal

Stimulation, monitoring, and oocyte retrieval were performed using a GnRH antagonist (Orgalutran, Merck Sharp, and Dohme, The Netherlands) protocol (6). When the mean diameter of at least two leading follicles was 17 mm (determined using ultrasound), ovulation was triggered with a subcutaneous bolus of 250 μg r-hCG (Ovitrelle, Merck Serono, Germany), with oocyte retrieval 36 h later.

### Early Luteal Phase Blood Sampling

Blood samples (2 mL) for determination of hormone levels were collected on the day of trigger, prior to the injection of hCG, at 12, 24, and 38 h after hCG injection, and at 1, 2, 3, 4, 5, and 6 days after oocyte pick-up (OPU). All samples were processed immediately and stored at −20°C. Serum hormone levels were determined using electrochemiluminescence immunoassay (ECLIA; Roche Cobas E 801, Roche Diagnostics, Germany) with a lower level of quantification of 0.1 mIU/mL and inter- and intra-assay variability of 2–5%, as described previously (6).

### Statistical Analysis

Data were analyzed using R version 3.3.3 software. All tests are two tailed, and *p* < 0.05 was considered statistically significant. Continuous variables are presented as mean ± standard deviation (SD) and were compared using the Student's *t*-test. Categorical data are expressed as numbers and were compared using the Chi-square test.

To determine whether there were any characteristics that predicted which patients might need additional luteal phase support, we compared patient and cycle characteristics in subjects with early luteal phase hCG values in the bottom 10% compared with patients whose serum hCG levels were in the remaining 90%. Correlations between potential predictive factors identified by comparing these patient subgroups were investigated for correlation with serum hCG level using the Spearman test.

To further define patient subgroups who could potentially benefit from increase luteal phase support, a cluster analysis was performed. Firstly, the K-mean method (k from 2 to 5) was used to group all patients with similar values for serum hCG and serum progesterone at the “hCG+12 h” timepoint until we observed a cluster that has low hCG and progesterone levels. Next we compared potential predictive characteristics for patients in this cluster compared with the other clusters and calculated the trend *p*-value.

## Results

### Patients

One hundred and sixty-one patients were initially enrolled in the study. Mean patient age was 31.8 ± 3.2 years (range 25–41 years), mean BMI was 20.5 ± 2.1 kg/m^2^ (range 15.6–27.1 kg/m^2^), mean AMH level was 4.58 ± 2.44 ng/mL (range 0.66–12.08 ng/mL), and mean AFC was 15.1 ± 6.3 (range 4–40). The main indications for IVF were male factor (38.5%), unexplained infertility (25.5%), and tubal factor (18.0%) After stimulation, there were 10.6 ± 4.6 follicles of ≥14 mm (range 0–23), 13.9 ± 6.0 oocytes (range 3–36), 11.3 ± 5.3 metaphase II oocytes (range 2–34), 6.4 ± 3.1 embryos (range 0–17), and 1 ± 1.3 good-quality embryos based on the Istanbul criteria (range 0–7) (8). The luteal phase duration was 10.9 ± 2.6 days (range 8–20).

### Inter-individual Variability in Serum hCG

Mean and median values for early luteal phase hCG concentrations showed wide variability around the mean and median values over the period from 12 h after hCG injection up to 6 days after oocyte pick-up (Table 1).

**Table 1:** Serum human chorionic gonadotropin (hCG) levels over time after a bolus dose of hCG to trigger ovulation (*n* = 161).

**Serum hCG (mIU/mL)**	**Minimum**	**Maximum**	**Mean ± SD**	**Median (IQR)**
Before hCG	0.00	12.32	0.35 ± 1.14	0.10 (0.1–0.1)
hCG+12 h	41.22	375.90	126.94 ± 52.49	118.40 (93.78–146.40)
hCG+24 h	41.32	327.50	131.53 ± 51.83	122.10 (97.44–154.80)
hCG+36 h	47.59	241.10	108.99 ± 34.82	106.00 (83.83–130.00)
OPU+1 day	24.17	158.80	64.39 ± 21.73	63.21 (50.22–78.38)
OPU+2 days	5.25	94.48	34.16 ± 13.49	32.56 (25.66–43.07)
OPU+3 days	2.91	49.73	17.28 ± 8.05	17.09 (12.51–21.99)
OPU+4 days	1.91	30.20	9.21 ± 4.81	8.23 (5.99–11.64)
OPU+5 days	0.71	17.17	5.03 ± 2.84	4.61 (3.19–6.34)
OPU+6 days	0.15	11.83	2.84 ± 1.91	2.43 (1.79–3.54)

*IQR, interquartile range; OPU, oocyte pick-up; SD, standard deviation*.

### Characteristics of Patients With Low Serum hCG After hCG Trigger

Patients with serum hCG values in the bottom 10% had a significantly higher BMI (*p* = 0.038) and a significantly longer duration of stimulation (*p* = 0.014) than those with higher serum hCG values (Table 2). All other patient characteristics and stimulation outcomes were similar in the two patient subgroups (Table 2). Serum progesterone levels up to the first 36 h after hCG injection were significantly higher in the low vs. higher serum hCG group, but were similar at all other time points (Table 3).

**Table 2:** Baseline, cycle and stimulation characteristics in subjects with the lowest 10% of early luteal phase human chorionic gonadotropin (hCG) values vs. patients with luteal phase hCG levels in the remaining 90%.

**Characteristic**	**Luteal phase serum hCG levels**		***p*-value**
	**Patients with values in the lowest 10% (*n* = 16)**	**Patients with values in the remaining 90% (*n* = 144)**	
Age, years	32.44 ± 3.72	31.76 ± 3.13	0.42
BMI, kg/m^2^	21.57 ± 2.48	20.41 ± 2.05	0.038
AMH, ng/mL	5.15 ± 2.59	4.48 ± 2.41	0.332
AFC, *n*	14.44 ± 6.45	15.10 ± 6.33	0.694
Indication for IVF, *n* (%):			0.686
Male factor	6 (37.5)	55 (38.2)	
Unexplained	5 (31.2)	24 (16.7)	
Tubal factor	4 (25.0)	37 (25.7)	
Ovulation disorder	1 (6.2)	8 (5.6)	
Diminished ovarian reserve	0 (0.0)	5 (3.5)	
Endometriosis	0 (0.0)	5 (3.5)	
Other	0 (0.0)	10 (6.9)	
Total dose of FSH used, IU/L	2648.44 ± 758.81	2350.87 ± 554.14	0.052
Duration of stimulation, days	9.31 ± 1.35	8.64 ± 0.99	0.014
Follicles ≥11 mm, *n*	14.07 ± 3.84	12.87 ± 4.39	0.312
Follicles ≥14 mm, *n*	10.00 ± 4.41	10.53 ± 4.55	0.66
Oocytes, *n*	15.94 ± 8.00	13.55 ± 5.65	0.127
MII oocytes, *n*	12.25 ± 6.36	11.15 ± 5.07	0.422
Embryos, *n*	6.25 ± 3.36	6.44 ± 3.13	0.822
Good embryos^*^, *n*	3.12 ± 1.67	3.69 ± 2.45	0.367
Frozen embryos^**^, *n*	4.25 ± 1.53	4.31 ± 1.95	0.912
OHSS, *n* (%)	0 (0.0)	1 (0.7)	

*Values are mean ± standard deviation, or number of patients (%)*.

*AFC, antral follicle count; AMH, anti-Müllerian hormone; BMI, body mass index; FSH, follicle-stimulating hormone; IVF, in vitro fertilization; MII, metaphase II; OHSS, ovarian hyperstimulation syndrome*.

* *Based on the Istanbul criteria (8)*.

** *As well as good embryos, some graded as fair (Istanbul criteria) were also frozen*.

**Table 3:** Early luteal phase serum progesterone levels in subjects with the lowest 10% of early luteal phase human chorionic gonadotropin (hCG) values vs. patients with luteal phase hCG levels in the remaining 90%.

**Serum progesterone, ng/mL**	**Luteal phase serum hCG levels**		***p*-value**
	**Patients with values in the lowest 10% (*n* = 16)**	**Patients with values in the remaining 90% (*n* = 144)**	
Before hCG	1.43 (0.70)	1.46 (1.11)	0.926
hCG+12 h	11.17 (5.21)	8.49 (4.47)	0.027
hCG+24 h	22.46 (14.12)	15.38 (7.09)	0.001
hCG+36 h	19.38 (10.45)	13.47 (6.31)	0.001
OPU+1 day	51.26 (13.95)	44.47 (15.81)	0.102
OPU+2 days	74.89 (27.94)	69.35 (25.56)	0.416
OPU+3 days	112.91 (54.94)	92.06 (41.23)	0.066
OPU+4 days	123.03 (39.73)	112.59 (49.52)	0.417
OPU+5 days	107.67 (52.66)	100.13 (53.43)	0.593
OPU+6 days	74.46 (63.26)	74.21 (47.60)	0.985

*Values are mean ± standard deviation*.

*OPU, oocyte pick-up*.

### Factors Correlated With Serum hCG Level

**Figure 1:**
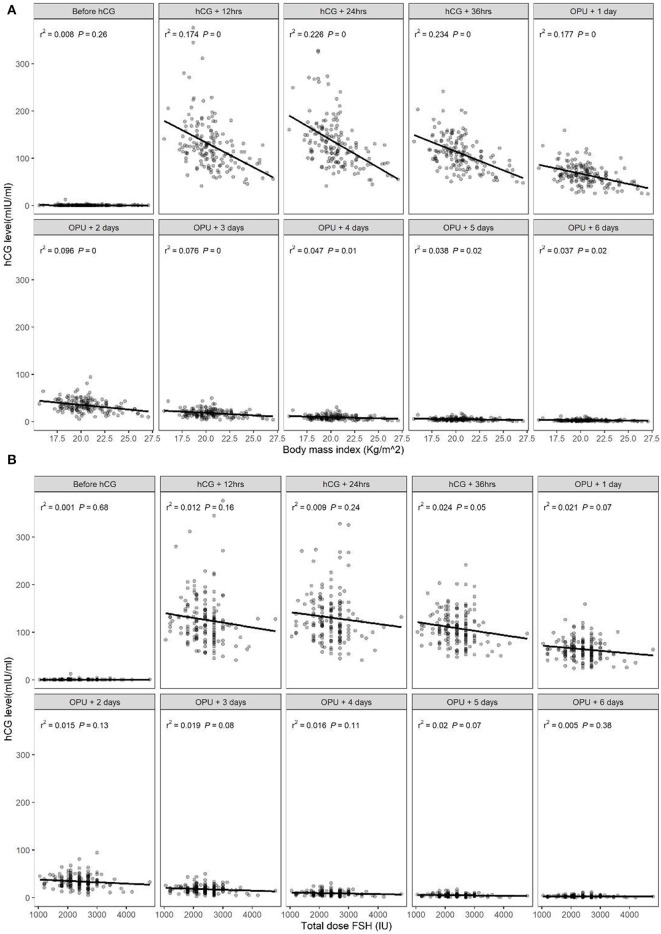
Relationship between serum human chorionic gonadotropin (hCG) levels in the early luteal phase and **(A)** body mass index (BMI) or **(B)** total dose of follicle-stimulating hormone (FSH).

There was a significant correlation between serum hCG level after hCG administration and BMI, with higher serum hCG levels in patients with a lower BMI (Figure 1A), consistent with the patient subgroup analysis. There was no significant association between serum hCG level and the total dosage of FSH (Figure 1B). There was also no significant correlation between peak hCG concentration and the number of follicles ≥14 mm, or between the time to reach peak hCG (i.e., peak hCG at 24 or 36 h after hCG or 1 day after OPU) and the number of follicles ≥14 mm (Table 4).

**Table 4:** Correlations between peak serum human chorionic gonadotropin (hCG) concentration and the number of follicles of ≥14 mm.

	**β** **(95% confidence interval)**			
	**Univariate linear regression**	***p*-value**	**Multivariate linear regression**	***p*-value**
Peak hCG value	−0.01 (−0.02, 0.01)	0.23	−0.01 (−0.02, 0.01)	0.23
**Peak hCG at:**				
hCG+24 h	−1.06 (−2.56, 0.43)	0.16	−1.12 (−2.62, 0.37)	0.14
hCG+36 h	0.1 (−2.72, 2.93)	0.94	−0.37 (−3.30, 2.55)	0.80
OPU+1 day	−1.06 (−7.51, 5.38)	0.75	−1.20 (−7.64, 5.24)	0.71

*OPU, oocyte pick-up*.

### Cluster Analysis

Patient characteristics by cluster are shown in Table 5. Patients in Cluster 1 who had significantly lower hCG and progesterone levels at 12 h after hCG injection also showed a significantly higher BMI, and significantly lower AMH, duration of stimulation and number of follicles of ≥11 and ≥14 mm (Table 5).

**Table 5:** Patient characteristics and stimulation outcomes by cluster.

**Characteristic**	**Cluster 1 (*n* = 31)**	**Cluster 2 (*n* = 28)**	**Cluster 3 (*n* = 61)**	**Cluster 4 (*n* = 40)**	**Trend *p*-value**
hCG at hCG+12 h	75.12 ± 17.33	100.10 ± 15.48	158.11 ± 60.81	138.40 ± 30.16	<0.001
Progesterone at hCG+12 h	5.02 ± 1.52	8.43 ± 1.16	13.05 ± 4.32	5.32 ± 1.69	<0.001
Age, years	33.10 ± 3.42	31.07 ± 2.52	31.54 ± 2.94	31.80 ± 3.60	0.071
BMI, kg/m^2^	22.10 ± 2.46	20.67 ± 1.88	19.91 ± 1.77	20.17 ± 1.93	<0.001
AMH, ng/mL	3.49 ± 2.01	4.88 ± 2.41	5.11 ± 2.29	4.24 ± 2.67	0.021
AFC, n	15.06 ± 6.83	14.39 ± 5.59	15.98 ± 6.70	14.00 ± 5.82	0.439
Total FSH dose, IU/L	2528.23 ± 604.42	2325.00 ± 556.40	2413.33 ± 613.71	2256.88 ± 519.77	0.237
Duration of stimulation, days	8.68 ± 1.08	8.71 ± 1.08	9.00 ± 0.98	8.28 ± 0.96	0.008
Follicles ≥11 mm, *n*	11.27 ± 3.50	13.11 ± 4.50	15.25 ± 3.85	10.92 ± 3.99	<0.001
Follicles ≥14 mm, *n*	9.42 ± 3.72	11.32 ± 4.39	11.82 ± 5.02	8.65 ± 3.63	0.002

*Values are mean ± standard deviation*.

*AFC, antral follicle count; AMH, anti-Müllerian hormone; BMI, body mass index; FSH, follicle-stimulating hormone; hCG, human chorionic gonadotropin; IVF, in vitro fertilization; MII, metaphase II; OHSS, ovarian hyperstimulation syndrome*.

## Discussion

This study demonstrated for the first time that there is pronounced variability in hCG concentrations during the early luteal phase following a subcutaneous bolus dose of hCG to trigger ovulation in women undergoing IVF. Therefore, a considerable number of patients are likely to experience low, and probably suboptimal, stimulation of their corpus luteum due to low concentrations of hCG. An important goal of the current analysis was to identify potential predictors of low hCG after a bolus trigger dose. This would allow adjustment of the dose of hCG to provide enough stimulation of the corpus luteum to produce progesterone for luteal phase support, increasing the chances of successful pregnancy. The observed association between serum hCG level after a bolus dose and BMI suggests that women with a higher BMI probably need additional luteal phase support because their hCG levels decline faster than in those with a lower BMI. This association was seen despite the fact that the study population from Vietnam had an overall relatively low BMI. It is therefore possible that the association between BMI and hCG levels would be even more relevant in Western or European populations who would have a higher BMI than patients in the current study.

The relationship between BMI and serum hCG levels is consistent with data from a previous study showing that obese women had significantly lower maximum serum concentration, area under the concentration-time curve and average hCG concentration after a subcutaneous injection of r-hCG compared with women who had a normal body weight (9). Comparison of patient characteristics and stimulation outcomes in subjects with the lowest 10% of serum hCG values vs. those with higher values from this analysis showed that it was difficult to predict in advance which patients would have low concentrations of hCG in the early luteal phase. It was hypothesized that when the number of follicles of ≥14 mm was greater, then consumption of hCG during the luteal phase would be higher. However, we did not find any association between peak serum hCG and the number of follicles of ≥14 mm suggesting that the hypothesis was not correct. Furthermore, the lack of association between time to peak hCG and the number of follicles of ≥14 mm indicates that consumption of hCG was not related to the number of corpora lutea. Therefore, luteal phase support does not need to be tailored based on the number of follicles. However, our data do suggest that some individualization of luteal phase support protocols might be beneficial. Current IVF clinical practice includes variations in the ovarian stimulation protocol based on patient characteristics, but all patients receive the same approach to luteal phase support.

The results of our cluster analysis suggest that patients with BMI > 22 kg/m^2^, AMH < 3.5 ng/mL, stimulation duration < 8.7 days, <11 follicles of ≥11 mm, and <9 follicles of ≥14 mm may be more likely to have low serum levels of hCG and progesterone at 12 h after hCG. Therefore, women with these characteristics might require a modified luteal phase support protocol to maximize the chances of achieving pregnancy. However, predicting patients who will experience low levels of hCG after a trigger dose is not simple, and how this should be approached in a clinical setting is not yet known. Perhaps it is worthwhile considering a small bolus injection of hCG (e.g., 1,000–1,500 IU) at 5 days after OPU, at least in cases where evaluation of hCG levels on the day of embryo transfer shows that these levels are only modest.

It is surprising that levels of progesterone were significantly higher in the group of women who experienced low levels of hCG throughout the observation period. This result is not readily explained but apparently the corpora lutea in the group of women with low hCG are more sensitive toward hCG.

The key limitation of this study is the retrospective nature of the analysis. However, this is the first time that there has been a detailed analysis of potential predictors of variations in early luteal phase level of hCG after administration of a bolus dose. The fact that we used only a recombinant form of hCG means that data are specific to this formation only, and a comparison between recombinant and urinary preparations is required. Another limitation is the single ethnicity population, and the relatively low overall BMI of the Vietnamese patients in this study compared with European or US women undergoing IVF. Therefore, the applicability of our findings to populations from other geographic regions remains to be determined. Finally, the effects of aging on serum hormone levels was not taken into account in our analysis. This could be a confounding factor given that our study population included patients aged from 25 to 41 years and there are age-related changes in the female hormonal environment during the reproductive years (10).

In conclusion, there is wide inter-individual variation in serum hCG levels after a single subcutaneous bolus trigger dose of r-hCG. BMI is one potential contributor to this variation, but more detailed information on the luteal phase hormonal profile and data on predictors of hormone levels during this critical period are needed. It is hoped that these data will contribute to the development of strategies to allow individualization of the luteal phase support regimen, contributing to optimization of outcomes in women undergoing IVF.

## Data Availability Statement

The raw data supporting the conclusions of this article will be made available by the authors, without undue reservation, to any qualified researcher.

## Ethics Statement

The studies involving human participants were reviewed and approved by My Duc Hospital Ethical Review Board. The patients/participants provided their written informed consent to participate in this study.

## Author Contributions

LV: study design, execution, analysis, manuscript drafting, critical discussion, and final approval of the manuscript. TP: data analysis, critical discussion, and final approval of the manuscript. VH: execution, critical discussion, and final approval of the manuscript. TH: study design, execution, critical discussion, and final approval of the manuscript. PH and CA: study design, critical discussion, and final approval of the manuscript.

### Conflict of Interest

The authors declare that the research was conducted in the absence of any commercial or financial relationships that could be construed as a potential conflict of interest. The reviewer AC declared a past co-authorship with authors PH and CA to the handling editor.
